# Effects of different soil water holding capacities on vegetable residue return and its microbiological mechanism

**DOI:** 10.3389/fmicb.2023.1257258

**Published:** 2023-09-07

**Authors:** Chao Lu, Qian Zhu, Meihua Qiu, Xinhui Fan, Jia Luo, Yonghong Liang, Yan Ma

**Affiliations:** ^1^Institute of Agricultural Resource and Environmental Sciences, Jiangsu Academy of Agricultural Sciences, Nanjing, China; ^2^National Agricultural Experimental Station for Agricultural Environment, Nanjing, China; ^3^Jiangsu Province Station of Farmland Quality and Agro-Environmental Protection, Nanjing, China

**Keywords:** water holding capacity, nitrogen form, vegetable residue, bacterial community, keystone species

## Abstract

With the gradual expansion of the protected vegetable planting area, dense planting stubbles and increasing labor cost, the treatment of vegetable residues has become an urgent problem to be solved. Soil bacterial community structure plays an important role in vegetable residue return and is susceptible to environmental changes. Therefore, understanding the influences of different soil water holding capacities on plant residue decomposition and soil bacterial communities is important for biodegradation. During the whole incubation period, the weight loss ratio of plant residue with 100% water holding capacity was 69.60 to 75.27%, which was significantly higher than that with 60% water holding capacity in clay and sandy soil, indicating that high water holding capacity promoted the decomposition of plant residue. The degradation of lignin and cellulose was also promoted within 14 days. Furthermore, with the increase in soil water holding capacity, the contents of NH_4_^+^ increased to 5.36 and 4.54 times the initial value in the clay and sandy soil, respectively. The increase in *napA* and *nrfA* resulted in the conversion of NO_3_^–^ into NH_4_^+^. The increase in water holding capacity made the bacterial network structure more compact and changed the keystone bacteria. The increase in water holding capacity also increased the relative abundance of Firmicutes at the phylum level and *Symbiobacterium*, *Clostridium* at the genus level, which are all involved in lignin and cellulose degradation and might promote their degradation. Overall, these findings provide new insight into the effects of different soil water holding capacities on the degradation of plant residues *in situ* and the corresponding bacterial mechanisms.

## 1. Introduction

With the continued improvement of people’s requirements for life, the types of vegetables cultivated and their planting areas have increased significantly, and the amount of vegetable residues with no commodity value has also increased (Ganesh et al., 2022). According to the estimated crop straw output, the amount of vegetable residue produced accounts for approximately 25.6% of all crop straws, reaching 230.4 million tons per year, and almost 45% of it was directly discarded (Mazareli et al., 2016; Yun et al., 2021). At present, the main modes of utilization of vegetable residue are *in situ* return, anaerobic digestion, and mixed aerobic composting (Ros et al., 2017; Cui et al., 2020). However, due to the characteristics of high moisture content, short storage time, and difficulty of transport, it is difficult to remove vegetable residues from the field for further treatment (Alvarez and Liden, 2008). Therefore, *in situ* return is regarded as an economical, convenient, and scalable method of disposal of vegetable residue.

Vegetable residue is rich in nitrogen and organic matter. It was reported to contain on average approximately 70% organic matter, 3% nitrogen, 0.84% phosphorus, and 2.46% potassium (Wei et al., 2021). The application of vegetable residue *in situ* has been proven to increase soil organic matter, improve water retention capacity, and stimulate microbial activity in greenhouses (Marousek et al., 2013), and the *in situ* return of vegetable residue was reported to increase soil fertility with fewer organic and chemical fertilizers (Wang et al., 2021b; Wei et al., 2021). However, due to its high moisture content, low carbon/nitrogen ratio, and the possibility of carrying harmful pathogens, there is little research on the application of vegetable residue return (Fernandez-Gomez et al., 2010; Pellejero et al., 2017). Nevertheless, due to the favorable control of pathogens in the planting process, *in situ* return of vegetable residue to the field has become gradually feasible, and thus more attention should be focused on it.

During the *in situ* straw return process, many factors, including soil moisture, pH, nitrogen and phosphorus content, and carbon/nitrogen (C/N) ratio can affect straw decomposition (Kamble and Baath, 2016; Zhao et al., 2019). It has been proven that the decomposition of crop straw, such as corn, rice, and wheat straw, can be accelerated by increasing the soil water content and applying nitrogen fertilizer (Li et al., 2006). Nitrogen addition, in particular, can stimulate microorganism reproduction and improve cellulose and lignin utilization efficiency by reducing the C/N ratio of soil (Meng et al., 2017). However, the input of nitrogen, phosphorus, and potassium to soil during vegetable planting has been very high, which is not suitable for promoting residue decomposition when adding additional nitrogen sources. The pathogenic microorganisms in the soil can also be killed by high-temperature stewing sheds. During the high-temperature stewing process, it is necessary to add water in advance. However, there are few studies on how to promote the degradation of vegetable residues in soil and the microbiological mechanism behind it when maintaining different soil water holding capacities during high-temperature stewing.

In this study, two soil types, clay and sandy soil, with three different soil water holding capacities (60, 80, and 100%) were selected. The composition of the vegetable residue of *Capsicum* L. and the physical and chemical properties of the soil were analyzed. Furthermore, Illumina high-throughput sequencing was used to analyze the dynamic changes in the bacterial community with different soil water holding capacities. Our objectives were to (1) determine whether the soil water holding capacities affect vegetable residue degradation; (2) identify the changes in soil physical and chemical properties with different soil water holding capacities; and (3) understand how the bacterial community adjusts to the different water holding capacities and determine their contribution to the changes in vegetable residues. Overall, our study will provide a theoretical reference for returning solanaceous vegetable straw to the field *in situ*.

## 2. Materials and methods

### 2.1. Soil sample and experimental design

The vegetable residue of *Capsicum* L. used in our study was taken from the pepper planting greenhouse in Luhe base (32°28′N, 118°37′E) of Jiangsu Academy of Agricultural Sciences, China. The clay soil tested was taken from the 0 to 25 cm topsoil layer of the vegetable greenhouse in Zhuzhen town, Nanjing, and the sandy soil tested was taken from the 0 to 25 cm topsoil layer of the vegetable greenhouse in Longpao street, Nanjing. These two soil types were first sieved through a 1-cm-mesh sieve in the field, bagged, taken back to the laboratory, and then sieved through a 0.85-mm-mesh sieve to remove plant roots and pebbles. The original physical and chemical properties of the tested clay and sandy soils were showed in Supplementary Table 1.

The dimensions of the test barrel were 22 cm × 22 cm × 30 cm. The straw addition amount was 1%. Each barrel was filled with 13 kg of soil, and according to the 1% addition amount, 130 g of vegetable residue was added to each barrel and mixed. Three net bags were buried in each barrel, each of which was filled with 1 kg of soil and 10 g of straw. The soil water capacity is adjusted to 60–100% by adding different amounts of water. In order to simulate the temperature change in the process of high-temperature stewing sheds, the temperature was simulated by the artificial climate box: 7:00–10:00 (35°C), 10:00–13:00 (45°C), 13:00–15:00 (50°C), 15:00–17:00 (45°C), 17:00–22:00 (35°C), 22:00–07:00 (30°C). In order to reduce the loss of water during incubation, the humidity of the incubator is controlled at 100%.

In the process of vegetable cultivation, the process of high-temperature stewing sheds is usually not more than 1 month. In our research, samples were collected on days 14, 21, and 28, respectively. Three net bags were randomly removed from each treatment. After the net bag was removed, the soil and vegetable residues were separated, and the residues were all removed and washed with water, dried, and weighed. The degradation rates of the vegetable residues were calculated according to the quality differences of vegetable residues before and after incubation. The soil samples were divided into two parts: one part was air-dried for determination of soil physical and chemical properties, and the other part was placed in a −80°C refrigerator for extraction of soil DNA and determination of bacterial community structure.

### 2.2. Soil physical and chemical analysis

Soil samples were extracted with 2M potassium chloride solution for the detection of NH_4_^+^-N and NO_3_^–^-N (Maharjan and Venterea, 2013). The soil organic matter (SOM) and total nitrogen (TN) were determined by an Element Vario MACRO cube elemental analyzer (Elementar, Germany). The total phosphorus (TP) and available phosphorus (AP) were determined by the molybdenum blue colorimetric method with a spectrophotometer. The total potassium (TK) and available potassium (AK) were determined by a BWB-XP flame photometer (BWB Technologies, UK). Soil mineral N was extracted with 2 M KCl solution in a 1:5 soil-solution ratio, and the NH_4_^+^-N and NO_3_^–^-N concentrations were determined by a Skalar San++ continuous flow analyzer (Skalar, Netherlands). The cellulose and lignin contents were determined as described in a previous study (Chen et al., 2019).

### 2.3. Soil total DNA extraction and high-throughput sequencing

Total DNA was extracted from approximately 0.20 g of soil collected on days 0, 14, and 28 and frozen at −80°C by using the DNeasy PowerSoil Kit (Qiagen, Germany). DNA concentrations were determined with a Nanodrop 2000c spectrophotometer (Thermo, USA). Then, the DNA was stored at −20°C for further high-throughput sequencing.

The following primers were used to amplify the V4-V5 region of the bacterial 16S rRNA gene: 515F (5′-GTGCCAGCMGCCGCGG-3′) and 907R (5′-CCGTCAATTCMTTTRAGTTT-3′). The 50-μL PCR mixture contained 2 ng of soil DNA, 25 μL TopTaq DNA Polymerase (Transgen, China), 1.0 μL of each primer and double-distilled water to adjust the final volume. The PCR was carried out on an ABI 2720 Thermal Cycler (Applied Biosystems, USA), and the PCR products were purified with Agencourt AMPure XP PCR Purification Beads (Beckman Coulter Ltd., UK). The purified PCR products were sequenced on an Illumina NovaSeq 6000 system (Genesky Biotechnologies Inc., China) according to the manufacturer’s recommendations.

### 2.4. Bioinformatics analysis

The amplicon sequence variant (ASV), which was inferred by DADA2, was used in our study. The alpha and beta diversities were used to evaluate differences in community complexity among the samples. The phylogenetic molecular ecological network of the bacterial community was analyzed by MENA,^1^ and Cytoscape was used for graphics (Zhou et al., 2010; Deng et al., 2012). Only the ASVs observed in more than 50% of samples were retained for subsequent network construction. The nodes and edges in the network represent the ASVs and the correlations between ASVs, respectively. The connectivity of each node was determined based on its within-module connectivity (Zi) and among-module connectivity (Pi). Canonical correlation analysis (CCA) and principal coordinates analysis (PCoA) were performed by R software with the vegan and ade4 packages. The PICRUSt2 were used to predict the metagenome functions. Correlation analysis and visualization were performed by R software with the vegan and corrplot packages.

### 2.5. Statistical analysis and accession numbers of high-throughput sequencing data

All data were processed with Microsoft Excel 2017 (Microsoft, Redmond, WA, USA). One-way ANOVA was conducted using SPSS 25.0 (IBM, Armonk, NY, USA). The data are the means of three replicates, while error bars indicate standard deviations. All the original sequence data were deposited in the Sequence Read Archive database under the accession number PRJNA898822.

## 3. Results

### 3.1. Dynamics of soil physical and chemical properties

**FIGURE 1:**
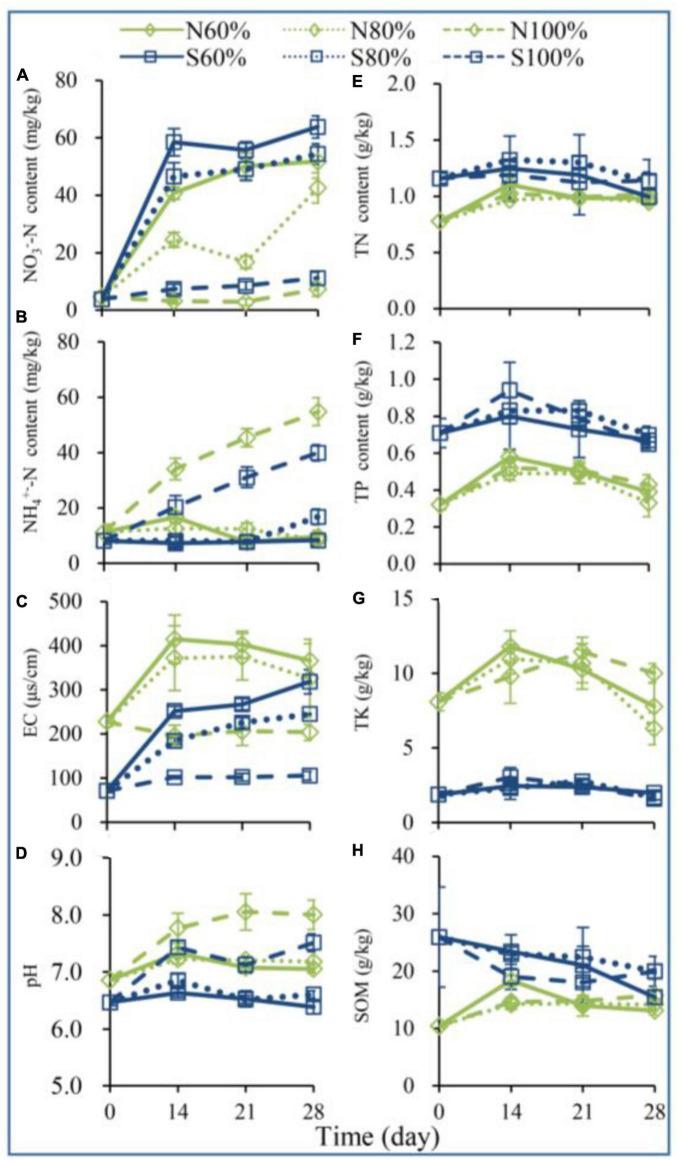
The contents of nitrate nitrogen (NO_3_^–^-N, **A**), ammonium nitrogen (NH_4_^+^-N, **B**), electric conductivity (EC, **C**), pH **(D)**, total nitrogen (TN, **E**), total phosphorus (TP, **F**), total potassium (TK, **G**), and total organic matter (SOM, **H**) in the soil. The N60, N80, and N100% refer to the clay soil with 60, 80, and 100% soil water holding capacities, respectively. The S60, S80, and S100% refer to the sandy soil with 60, 80, and 100% soil water holding capacities, respectively.

To explore the changes in soil physical and chemical properties during vegetable residue decomposition, the contents of SOM, TN, TP, TK, AP, AK, NH_4_^+^-N, and NO_3_^–^-N were detected over 28 days. With the increase in soil water holding capacity, the soil pH, NH_4_^+^-N, and NO_3_^–^-N changed, and the changes in NH_4_^+^-N and NO_3_^–^-N contents showed different trends (Figures 1A, B). On day 28, the NH_4_^+^-N contents of N100% and S100% were 54.82 mg/kg soil (which was 5.36 times that of N60%) and 39.96 mg/kg soil (which was 4.54 times that of S60%), respectively. On day 28, the NO_3_^–^-N contents of N100% and S100% were 7.35 mg/kg soil (which was 14.24% of that of N60%) and 11.17 mg/kg soil (which was 17.50% of that of N60%), respectively.

As shown in Figures 1F, G and Supplementary Figure 1, after 28 days of incubation, both in clay and sandy soil, the changes in soil water holding capacity from 60 to 100% had no significant effect on TP, TK, AP, and AK. As shown in Figure 1D, on day 28, the pH of N100% was significantly higher than that of N60% (ANOVA, *P* < 0.05), and a similar result was also observed in S60 and S100%.

### 3.2. Changes in biomass of plant residues

**FIGURE 2:**
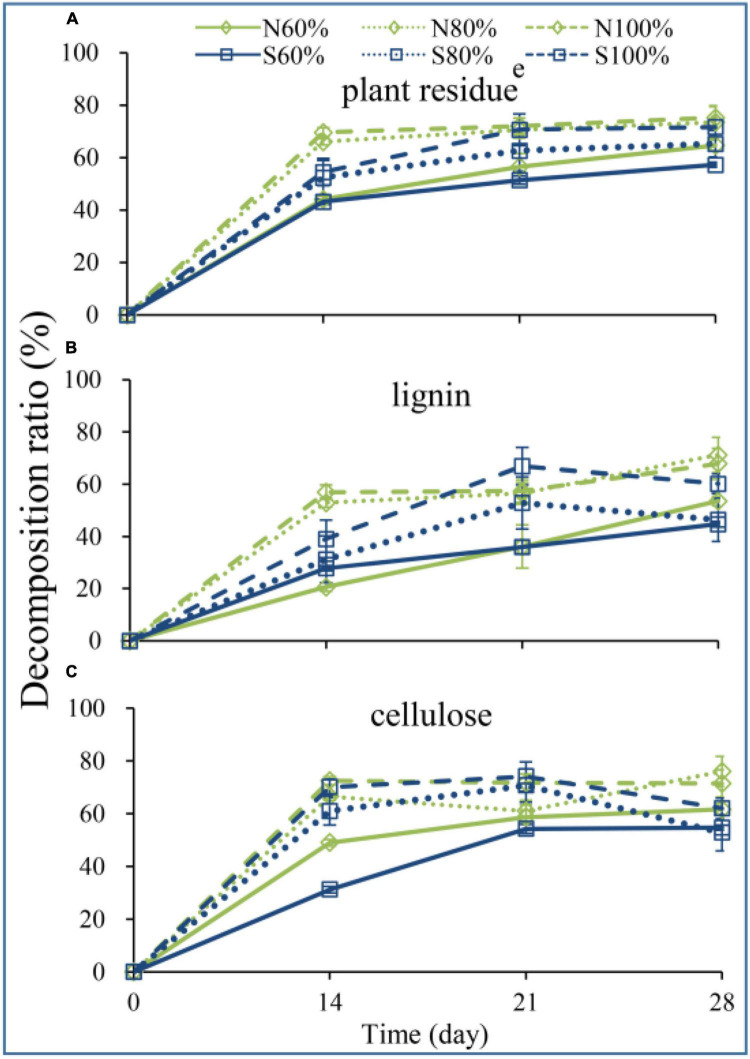
The decomposition ratio of plant residue **(A)**, lignin **(B)**, and cellulose **(C)**. The N60, N80, and N100% refer to the clay soil with 60, 80, and 100% soil water holding capacities, respectively. The S60, S80, and S100% refer to the sandy soil with 60, 80, and 100% soil water holding capacities, respectively.

To explore the decomposition of vegetable residue, the weight, lignin and cellulose contents of the residue were detected during incubation. As shown in Figure 2A, the residue weight loss rates of N80 and N100% on days 14, 21, and 28 were all significantly higher than those of N60% (ANOVA, *P* < 0.05), reaching 66.08%, 70.53%, 73.47%, and 69.60%, 72.03%, 75.27%, respectively. In sandy soil, the residue weight loss rates of S100% on days 14, 21, and 28 were significantly higher than those of S60% (ANOVA, *P* < 0.05), reaching 54.49, 70.63, and 71.63%, respectively. Furthermore, increasing the soil water holding capacity improved the degradation efficiency of lignin at the initial incubation stage and improved the final degradation rate. As shown in Figure 2B, the degradation rates of lignin of N80 and N100% on day 14 were 52.98 and 57.02%, respectively, which were significantly higher than those of N60% (ANOVA, *P* < 0.05). However, the degradation rate of lignin of S100% on day 14 was 39.11%, which was significantly higher than that of N60% (ANOVA, *P* < 0.05). Similar results were also observed for cellulose degradation. The degradation rates of cellulose of N100% on days 14 and 28 were 72.46 and 71.43%, respectively, which were significantly higher than those of N60% (ANOVA, *P* < 0.05). Additionally, the degradation rates of cellulose of S100% on days 14 and 28 were 70.05 and 61.97%, respectively, which were significantly higher than those of S60% (ANOVA, *P* < 0.05).

### 3.3. Bacterial community changes with different soil water holding capacities

Illumina high-throughput sequencing was used to evaluate the effect of different soil water holding capacities on the bacterial community. DNA was extracted and sequenced from the samples on days 0, 14, and 28. As shown in Table 1 and Supplementary Table 2, on day 14, there was no significant difference in the alpha diversity of clay and sandy soils with different soil water holding capacities. The Observed ASVs, Chao1, ACE, and Shannon indexes ranged from 2205.33 to 2410.33, 2231.19 to 2447.30, 2227.80 to 2438.36, and 6.77 to 7.04 in clay soil, respectively, and ranged from 2484.33 to 2769.67, 2514.90 to 2818.42, 2515.70 to 2811.90, and 6.95 to 7.17 in sandy soil, respectively. However, on day 28, compared with the treatments with 60% soil water holding capacity in sandy soil (S60%-28), the treatment with 100% soil water holding capacity (S100%-28) significantly enhanced the alpha diversity according to Observed ASVs, Chao1, ACE, and Shannon indexes (ANOVA, *P* < 0.05). As shown in Supplementary Figure 2, from days 14 to 28, the number of common ASVs of the three treatments gradually decreased, and the number of unique ASVs gradually increased.

**TABLE 1:** The alpha index of bacterial community.

	Observed ASV	Chao1	ACE	Shannon		Observed ASV	Chao1	ACE	Shannon
N60%-14	2,410.33a	2,447.3a	2,438.36a	7.04a	S60%-14	2,484.33a	2,514.90a	2,515.70a	6.95a
N80%-14	2,384.67a	2,417.8a	2,413.16a	7.00a	S80%-14	2,769.67a	2,818.42a	2,811.90a	7.17a
N100%-14	2,205.33a	2,231.19a	2,227.8a	6.77a	S100%-14	2,551.00a	2,585.60a	2,579.72a	7.03a
N60%-28	2,327.33ab	2,370.42ab	2,355.91ab	7.02a	S60%-28	2,218.67b	2,246.46b	2,247.95b	6.81b
N80%-28	2,439a	2,475.02a	2,466.22a	7.05a	S80%-28	2,466.67a	2,504.61a	2,499.45a	6.99a
N100%-28	2,096.67b	2,123.68b	2,116.98b	6.68b	S100%-28	2,565.33a	2,605.86a	2,599.81a	7.04a

**FIGURE 3:**
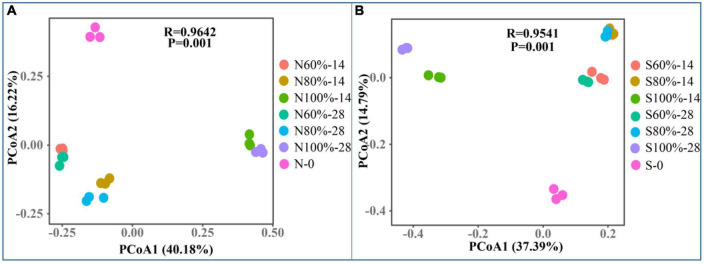
The principle coordinates analysis of clay **(A)** and sandy **(B)** soil with different soil water holding capacities. The N60, N80, and N100% refer to the clay soil with 60, 80, and 100% soil water holding capacities, respectively. The S60, S80, and S100% refer to the sandy soil with 60, 80, and 100% soil water holding capacities, respectively. The number following the percentage indicates the number of days.

Beta diversity was used to evaluate differences in the community complexity among samples and was calculated by principal coordinates analysis (PCoA). As shown in Figures 3A, B, with the increase in soil water holding, the bacterial community gradually evolved. In clay soil, on the PCo1 axis, the difference between N100%-14 and N60%-14 was greater than that between N80%-14 and N60%-14, and the same result was observed on day 28. Furthermore, the same phenomenon was observed in sandy soils, indicating that 100% soil water holding capacity had a greater impact on the bacterial community.

### 3.4. Bacterial community changes at the phylum and genus levels

**FIGURE 4:**
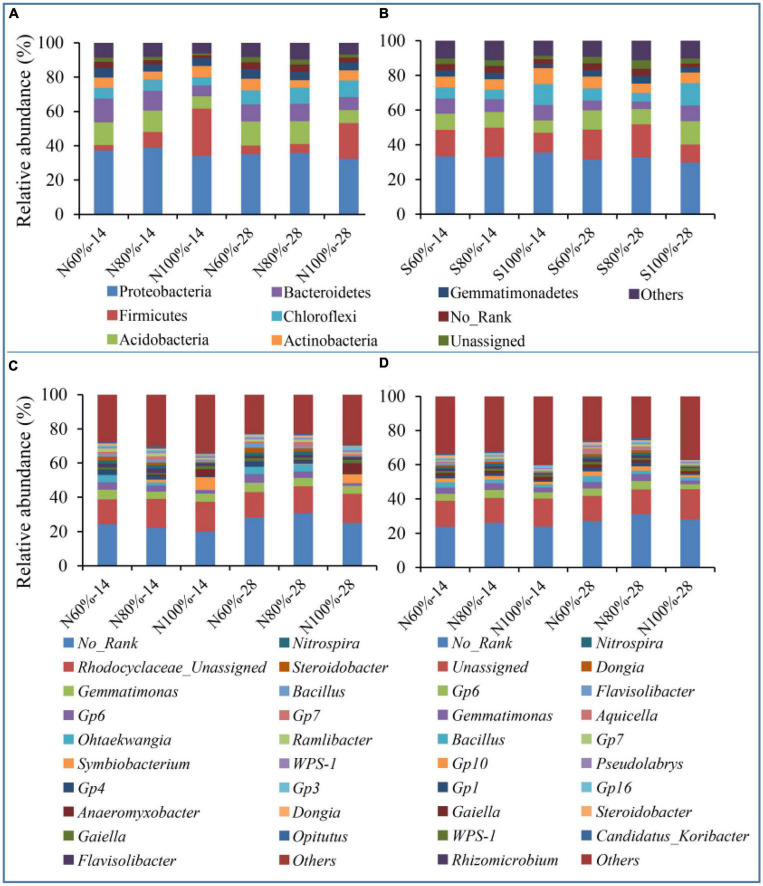
Relative abundance of bacterial species at the phylum and genus levels. **(A)** The relative abundance of the top10 bacterial phylum in clay soil. **(B)** The relative abundance of the top10 bacterial phylum in sandy soil. **(C)** The relative abundance of the top20 bacterial genus in clay soil. **(D)** The relative abundance of the top20 bacterial genus in sandy soil. The N60, N80, and N100% refer to the clay soil with 60, 80, and 100% soil water holding capacities, respectively. The S60, S80, and S100% refer to the sandy soil with 60, 80, and 100% soil water holding capacities, respectively. The number following the percentage indicates the number of days.

To study the effects of the different treatments on the bacterial community, we analyzed the changes in bacterial community structure at the phylum and genus levels. As shown in Figures 4A, B, the main bacterial strains at the phylum level were Proteobacteria (34.65% on average), Firmicutes (10.52% on average), and Acidobacteria (11.75% on average) and these three phyla accounted for approximately 56.92% of bacteria in the clay soil. In the sandy soil, the main bacterial strains at the phylum level were Proteobacteria (32.88% on average), Acidobacteria (14.60% on average), and Chloroflexi (9.58% on average), and these three phyla accounted for approximately 57.07% of the total bacteria. At the genus level, as shown in Figures 4C, D, *Rhodocyclaceae_Unassigned* (15.41%), *Gemmatimonas* (4.81%), *Gp6* (3.70%), *Ohtaekwangia* (2.44%), and *Symbiobacterium* (2.10%) were the main bacterial genera in the clay soil, and *Gp6* (4.05%), *Gemmatimonas* (3.49%), *Bacillus* (2.32%), and *Gp10* (2.05%) were the main bacterial genera in the sandy soil.

With the increase in soil water holding capacity, the relative abundance of Firmicutes increased significantly. The relative abundances of Firmicutes in N100%-14 and N100%-28 were 27.52 and 20.85%, respectively, which were significantly higher than those in N60%-14 (3.07%) and N60%-28 (4.98%), respectively (ANOVA, *P* < 0.05). In regard to the sandy soil, with the increase in soil water holding capacity, the relative abundances of Firmicutes in S100%-14 and S100%-28 were 12.13 and 12.97%, respectively, which were significantly higher than those in S60%-14 (6.34%) and S60%-28 (6.95%), respectively (ANOVA, *P* < 0.05).

At the genus level, in the clay soil, the relative abundances of *Symbiobacterium* in N100%-14 and N100%-28 were 7.46 and 5.21%, respectively, and those of *Clostridium* in N100%-14 and N100%-28 were 1.53 and 1.04%, respectively. In the sandy soil, the relative abundances of *Clostridium* in S100%-14 and S100%-28 were 1.14 and 1.02%, respectively. Both of these two genera belong to Firmicutes, and their relative abundances in the treatments with 100% soil water holding capacity were significantly higher than those with 60% soil water holding capacity (ANOVA, *P* < 0.05).

### 3.5. Relationships among the bacterial community, physical, and chemical properties in the soils

**FIGURE 5:**
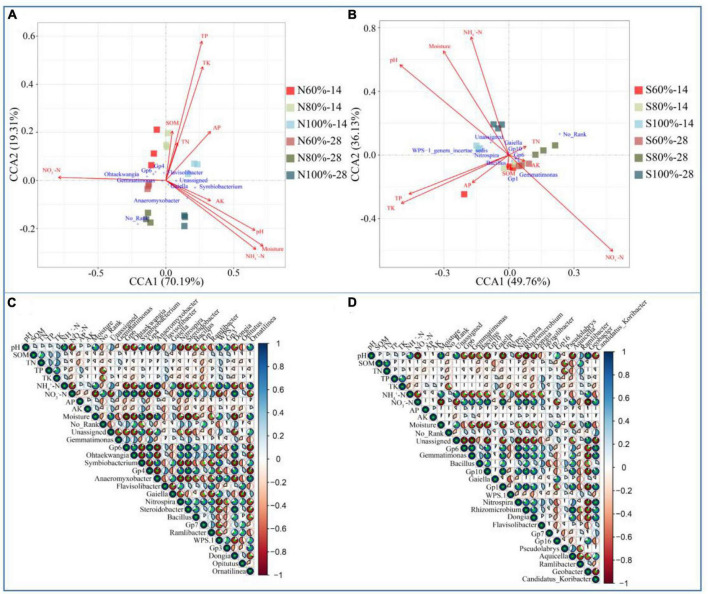
Canonical correlation analysis of bacterial community at the genus level in clay **(A)** and sandy **(B)** soil and Pearson’s correlation analysis among soil properties and bacteria at the genus level in clay **(C)** and sandy **(D)** soil.

To understand the effects of environmental factors on the bacterial community, CCA was used in our study. As shown in Figure 5A, the CCA1 axis and CCA2 axis explained 70.19 and 19.31% of the variation in the clay soil, respectively, and as shown in Figure 5B, the CCA1 axis and CCA2 axis explained 49.76 and 36.13% of the variation in the sandy soil, respectively. Whether in sandy or clay soil, the angles between NO_3_^–^-N and pH, NO_3_^–^-N and NH_4_^+^-N, NO_3_^–^-N and moisture were all larger than less than 90°. The projections of the three vectors of pH, NH_4_^+^-N, and moisture on the reverse extension line of the NO_3_^–^-N vector were longer than those of others. Pearson’s correlation analysis was used to further analyze the significance of the correlation among different environmental factors. As shown in Figures 5C, D, in both clay and sandy soils, the NO_3_^–^-N content was significantly negatively correlated with moisture, NH_4_^+^-N content, and pH (*P* < 0.01).

### 3.6. Bacterial phylogenetic molecular ecological network

**FIGURE 6:**
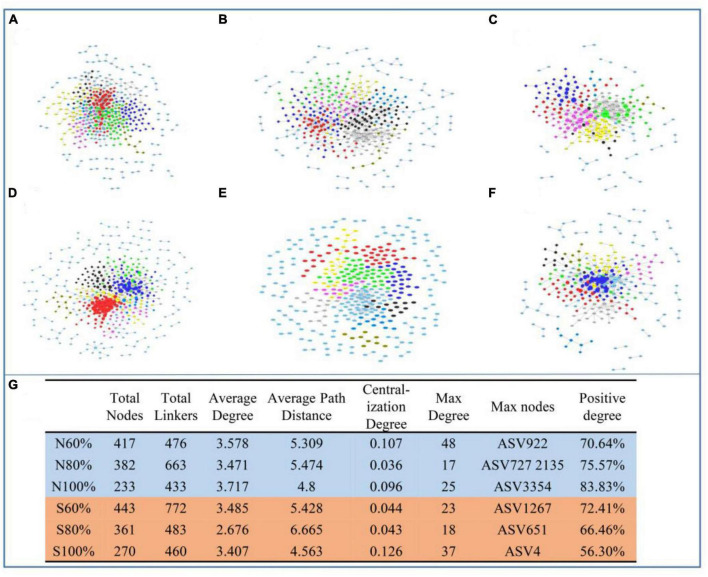
Ecological network analysis of bacterial community with different soil water holding capacities in soil and the detailed parameters of networks. The networks in clay soil with 60% **(A)**, 80% **(B)**, and 100% **(C)** soil water holding capacities and in sandy soil with 60% **(D)**, 80% **(E)**, and 100% **(F)** soil water holding capacities. **(G)** The detailed parameters of the networks.

To discern the co-occurrence patterns of the soil bacterial community with different soil water holding capacities, molecular ecological networks were constructed in our study. With the increase in soil water holding capacity, the number of nodes and links of the networks decreased. As shown in Figures 6A–C, there were 417, 382, 233 nodes and 476, 663, 433 links in the networks of the treatments with 60, 80, and 100% soil water holding capacity in the clay soil, respectively. As shown in Figures 6D–F, in sandy soil, there were 443, 361, 270 nodes and 772, 483, 460 links in the networks, respectively. The maximum node degrees of the treatments with 60, 80, and 100% field water capacity in the clay soil were 48, 17, and 25, respectively, and the maximum nodes were ASV922 (*Nitrospira*), ASV727 (*WPS-1*) and ASV2135 (*Gaiella*), and ASV3354 (*Erythrobacteraceae*), respectively. In the sandy soil, the maximum node degrees of the treatments with 60, 80, and 100% soil water holding capacity in the clay soil were 23, 18, and 37, respectively, and the maximum nodes were ASV1267 (*Elusimicrobium*), ASV651 (*Myxococcales*), and ASV4 (*Prolixibacteraceae*), respectively. In both types of soil, the average path distances at 100% water capacity were smaller than those at 60% water capacity, indicating that the relationships among communities were closer in the soil with higher water capacity (Figure 6G).

In the networks, the network hub, connector, and module hub to which the ASVs belong were regarded as the keystone species. The within-module connectivity (Zi) and among-module connectivity (Pi) were calculated in our study. As shown in Table 2 and Supplementary Figure 4, in the clay soil, there was no network hub with Zi > 2.5 and Pi > 0.62 in any network. However, in the clay soil, there were 10, 8, and 3 module hubs with Zi > 2.5 in the networks with 60, 80, and 100% soil water holding capacity, respectively, and there were 18, 10, and 6 connectors with Pi > 0.62 in the treatments with 60, 80, and 100% soil water holding capacity, respectively.

**TABLE 2:** The module hubs and connectors in the networks.

	Module hubs	Connectors
N60%	ASV922, 2,534, 2,340, 1,554, 718, 99, 589, 3,466, 2,097, 401	ASV25, 76, 836, 115, 4,224, 3,099, 3,040, 249, 1,786, 853, 2,339, 93, 355, 636, 140, 726, 502, 2,403
N80%	ASV316, 1,598, 280, 857, 424, 478, 108, 717	ASV440, 425, 260, 1,237, 355, 704, 1,000, 502, 685, 808
N100%	ASV532, 2,362, 737	ASV2730, 3,354, 1,830, 302, 344, 71
S60%	ASV2643, 859, 1,850, 576, 3,925, 1,188, 304	ASV2618, 1,283, 1,072, 1,370, 1,697, 413, 1,850, 1,411, 870, 696, 724
S80%	ASV347, 2,035, 3,798, 2,054, 859, 1,806	ASV875, 304, 122
S100%	ASV4, 1,407, 673, 714, 744, 79	ASV71, 2,734, 259, 1,966, 1,758, 226

In the sandy soil, there were 7, 6, and 6 module hubs in the networks in the treatments with 60, 80, and 100% soil water holding capacity, respectively. There were 11, 3, and 6 connectors in the treatments with 60, 80, and 100% soil water holding capacity, respectively. In the module hubs and connectors, ASV1850 (*Mizugakiibacter*), for which Zi > 2.5 and Pi > 0.62, was considered to be the network hub of networks in the treatment with 60% soil water holding capacity.

## 4. Discussion

### 4.1. Soil water holding capacity affects the decomposition of plant residues

In process of straw return to the field, the moisture content of straw determines its decomposition rate. Some studies have confirmed that with the increase in moisture contents in rice and maize straw, the degradation of straw is promoted (Sain and Broadbent, 1975; Reddy et al., 1994, 2009; Thongjoo et al., 2005). Additionally, studies have found that the rate and extent of straw decomposition increase and that the lag period decreases with increasing relative humidity and temperature (Sain and Broadbent, 1975; Reddy et al., 1994). In our study, from day 14 to day 28, whether in clay or sandy soil, the weight loss rate of plant residue in the treatment with 60% soil water holding capacity was significantly lower than that in the treatment with 80 or 100% soil water holding capacity, indicating that high soil water holding capacity promoted straw degradation (Figure 2). Similar to our results, increasing the water content also promoted the degradation of lignin and cellulose and enhanced the soil organic matter content (Donnelly et al., 1990; Madadi and Abbas, 2017). On the one hand, with the increase in soil water holding capacity, although the degradation rate of straw was significantly increased, it had no significant impact on the physical and chemical properties of the soil, including TP, TK, AP, and AK. On the other hand, the pH, NH_4_^+^-N, and NO_3_^–^-N changed significantly.

### 4.2. Effects of nitrogen content and form on straw degradation

The C/N ratio is regarded as one of the important factors affecting the decomposition of plant straw (Nicolardot et al., 2001; Liang et al., 2017). Generally, the most suitable C/N ratio for the microbial degradation of cellulose and lignin is approximately 25 (Azim et al., 2018). However, in the planting process of facility soil, the content of N in the soil is usually high, which may lead to a low C/N ratio. In our study, the range of nitrogen content in the soil was 0.78 to 1.32 g/kg, and the range of the C/N ratio was 7.77 to 12.96, indicating that the soil-provided nitrogen was able to meet the requirements for microbial straw degradation. Similar to our study, the degradation of vegetable residues was usually fast during vegetable planting in facility soils with higher nitrogen content (Cao et al., 2018). Some studies have confirmed that straw decomposition can be accelerated by adding a certain amount of nitrogen fertilizer. Henriksen and Breland (1999) found that the content of available nitrogen in soil was one of the important factors limiting the decomposition of straw. However, in the cultivation process of greenhouse vegetables such as tomato and pepper, traditional farming methods have made the nitrogen content in the soil high. As shown in Figure 1, the TN, NH_4_^+^-N, and NO_3_^–^-N in clay soil were 0.78 g/kg, 11.25 mg/kg, and 4.54 mg/kg, respectively, and in sandy soil were 1.16 g/kg, 8.11 mg/kg, and 3.11 mg/kg, respectively. The total amount of ammonium and nitrate nitrogen was sufficient for the microbial degradation of straw. Thus, nitrogen content is not the limiting factor hindering the degradation of plant residues.

However, there have been few studies on the nitrogen forms required for the decomposition of plant residues. In our study, we found that with the degradation of residue, the content of NH_4_^+^-N in the soil gradually increased in the treatments with 80 and 100% soil water holding capacity. Correlation analysis found that the content of NH_4_^+^-N in the soil was significantly positively correlated with the weight loss rate of the residue and the degradation rates of cellulose and lignin (Figures 5C, D). At the same time, CCA indicated that the increase in the content of NH_4_^+^-N was an important factor promoting the decomposition of plant residue. However, it has been reported that high-quality straw (low C/N ratio) could cause a higher nitrogen mineralization rate and release of inorganic nitrogen during straw return to the field.

However, in our study, the total amounts of NH_4_^+^-N and NO_3_^–^-N in the different treatments did not change significantly, but the proportions of NH_4_^+^-N and NO_3_^–^-N did change significantly. For example, on day 28, the contents of NH_4_^+^-N in different soil types with 100% soil water holding capacity were significantly higher than those in the corresponding soil with only 60% soil water holding capacity. Through functional prediction, we found that the higher the soil water holding capacity of the soil was, the higher the relative abundance of genes involved in the dissimilatory reduction of nitrate to ammonium, such as *napA* and *nrfA*, which constitute a complete pathway for the conversion from nitrate to ammonium (Supplementary Figure 3; Smith et al., 2007). The increase in the two genes that constitute a complete pathway for the conversion from nitrate to ammonium might be the reason for the change in nitrate and ammonium content. At the same time, many studies have revealed that the higher the soil water content is, the higher the denitrification in the soil (Heinen, 2006). However, in this experiment, whether the root cause of ammonium nitrogen increase was the dissimilatory reduction of nitrate nitrogen or other processes needs further study.

### 4.3. Microbial response to plant residues in soil

Microorganisms are the main driving force of straw decomposition in the soil. Many different bacterial genera, such as *Bacillus*, *Streptomyces*, *Cellulomonas*, and *Cytophaga*, have been confirmed to be involved in straw degradation (Mason et al., 2001; Xinfeng et al., 2017; Hegazy et al., 2018; Spertino et al., 2018). In our study, at the phylum level, with the increase in soil water holding capacity, the relative abundance of Firmicutes in clay and sandy soil gradually increased (Figures 4A, B). To our knowledge, Firmicutes was reported as one of the most important phyla that can breakdown lignin and cellulose into sugars and contains a few cellulose-degrading bacteria, such as *Butyrivibrio*, *Clostridium*, and *Ruminococcus* (Liang et al., 2020; Wang et al., 2021a). The propagation of Firmicutes caused by the increase in soil water holding capacity was the main cause of straw degradation in our study, which was also observed in previous studies (Cheng et al., 2017; Gavande et al., 2021).

At the genus level, whether in clay or sandy soil, the relative abundance of *Symbiobacterium*, *Clostridium_sensu_stricto*, and *Clostridium_III* increased with increasing soil water holding capacity (Figures 4C, D). The increase in the abundance of the above cellulose-degrading bacteria was one of the reasons for the accelerated degradation of cellulose. Furthermore, *Clostridium*, which has been identified to harbor the *napA* and *nrfA* genes, is regarded as one of the main bacteria involved in the dissimilatory reduction of nitrate to ammonium (Braun et al., 1996; Li et al., 2018). In our study, the relative abundance of *Clostridium* increased with increasing soil water holding capacity.

The changes in soil water holding capacity also changed the structure of the bacterial network during the degradation of plant residues. Due to the importance of keystone bacteria, including module hubs, connectors, and network hubs, a few studies have focused on the identification of keystone bacteria in soil, plant, and human microbiomes (Banerjee et al., 2018; Tipton et al., 2018). In our study, compared with the 60% water holding capacity soil, the soils with the higher soil water holding capacities harbored distinct keystone bacteria, and the amounts of module hubs and connectors were all higher than those in soil with 60% soil water holding capacity. Usually, resources and energy are regarded as the main factors driving microbial community change (Foster et al., 2012). In our study, it was hence found that with the increase in soil water holding capacity and ammonium nitrogen content, the bacterial community tended to use cellulose or lignin from straw as a carbon source. Furthermore, the bacterial community assembly of the soils with 80 and 100% soil water holding capacities were simpler than that in soil with 60% soil water holding capacity in both clay and sandy soil. The simpler bacterial community assembly may be for a more singular and powerful function. In our research, with 100% soil water holding capacity, the keystone species, including *Mycobacterium* and *Melioribacter*, were both skilled at cellulose or lignin degradation.

Previous studies have confirmed that community complexity is closely related to community function. Complex communities have diverse functions and are well adapted to the environment, while simple communities tend to have a certain function (Niu et al., 2017; He et al., 2018). The addition of a recombinant simple microbial community could play a specific role in soil, especially in the control of soil-borne diseases (Li et al., 2021). In our study, the simpler microbial network structure may cause the community to tend to degrade the plant residue.

## 5. Conclusion

With the transformation of nitrate nitrogen in soil to ammonium nitrogen, high soil water holding capacity promoted the decomposition of plant residues in soil. The bacterial community also became more conducive to the degradation of cellulose and lignin, which are the main components of straw. At the genus level, the increasing abundance of *Clostridium* and *Symbiobacterium* might play a key role in the degradation of cellulose and lignin. Thus, it is anticipated that these results would benefit the development of agricultural waste treatment.

## Data availability statement

The datasets presented in this study can be found in online repositories. The names of the repository/repositories and accession number(s) can be found in this article/Supplementary material.

## Author contributions

CL: Investigation, Writing – original draft, Visualization. QZ: Investigation, Data curation. JL: Investigation, Data curation. MQ: Funding acquisition, Conceptualization. XF: Funding acquisition, Conceptualization. YL: Funding acquisition, Conceptualization. YM: Funding acquisition, Investigation, Data curation.

## Footnotes

1
http://ieg4.rccc.ou.edu/mena/main.cgi
