# Supplementary material for: Effects of different soil water holding capacities on vegetable residue return and its microbiological mechanism
Source: Front Microbiol. 2023 Sep 7;14:1257258. doi: 10.3389/fmicb.2023.1257258 (PMC10513456; doi:10.3389/fmicb.2023.1257258)
Supplement: Supplementary file 1 [file Data_Sheet_1.docx]

Effects of different soil water holding capacities on vegetable residue return and its microbiological mechanism

Chao Lu^1,2^, Qian Zhu^1,2^, Meihua Qiu^3^, Xinhui Fan^3^, Jia Luo^1,2^, Yonghong Liang^3,^*, Yan Ma^1,2^

1. Institute of Agricultural Resource and Environmental Sciences, Jiangsu Academy of Agricultural Sciences, Nanjing 210014, China

2. National Agricultural Experiment Station for Agricultural Environment, Luhe, Nanjing 210014, China;

3. Jiangsu Province Station of Farmland Quality and Agro-Environmental Protection，Nanjing 210036, China

*Corresponding author:

Yonghong Liang, E-mail: [1597152683@qq.com](mailto:1597152683@qq.com)

Yan Ma, E-mail: myjaas@sina.com


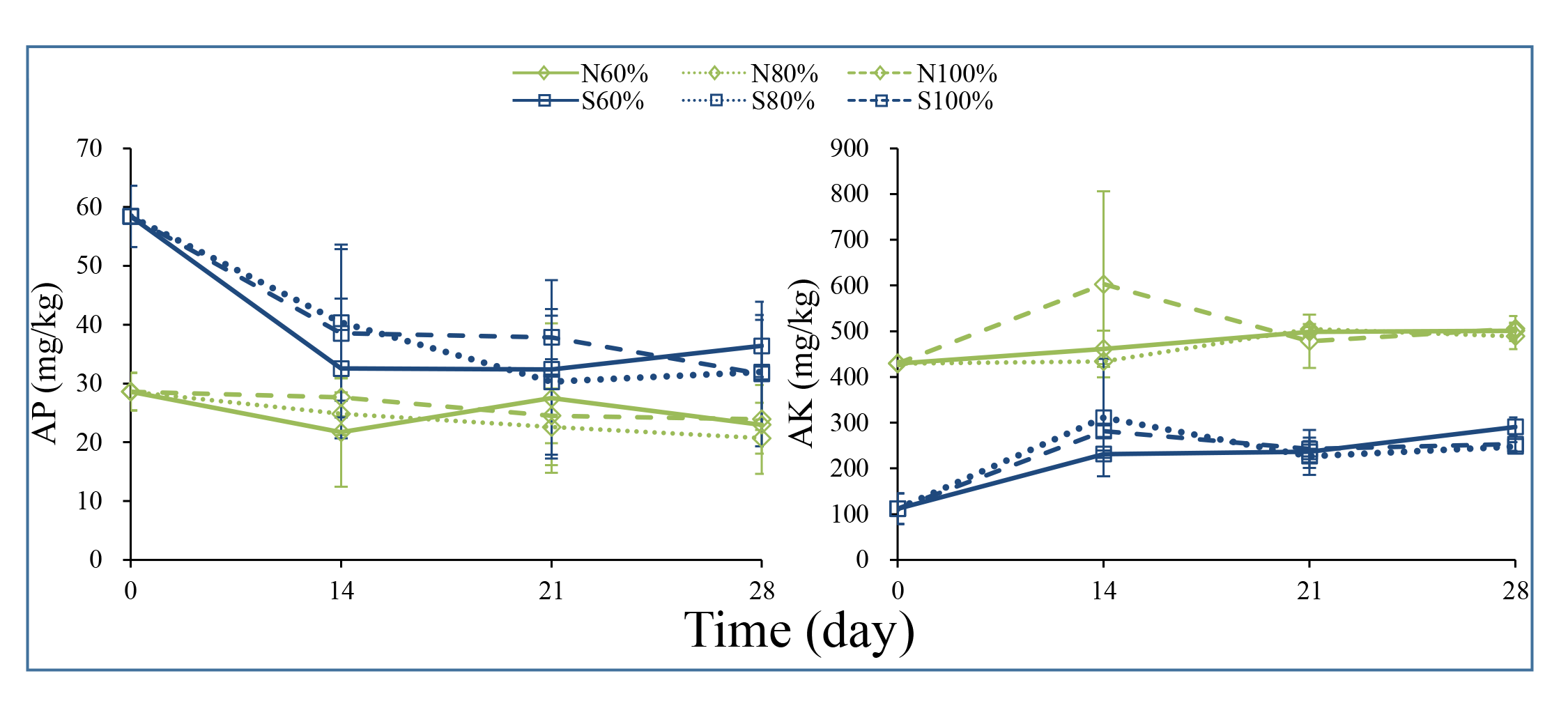


Figure S1 The contents of available phosphorus (AP) and available potassium (AK) in the soils. The N60%, N80%, and N100% refer to the clay soil with 60%, 80%, and 100% filed water capacities, respectively. The S60%, S80%, and S100% refer to the sandy soil with 60%, 80%, and 100% filed water capacities, respectively.


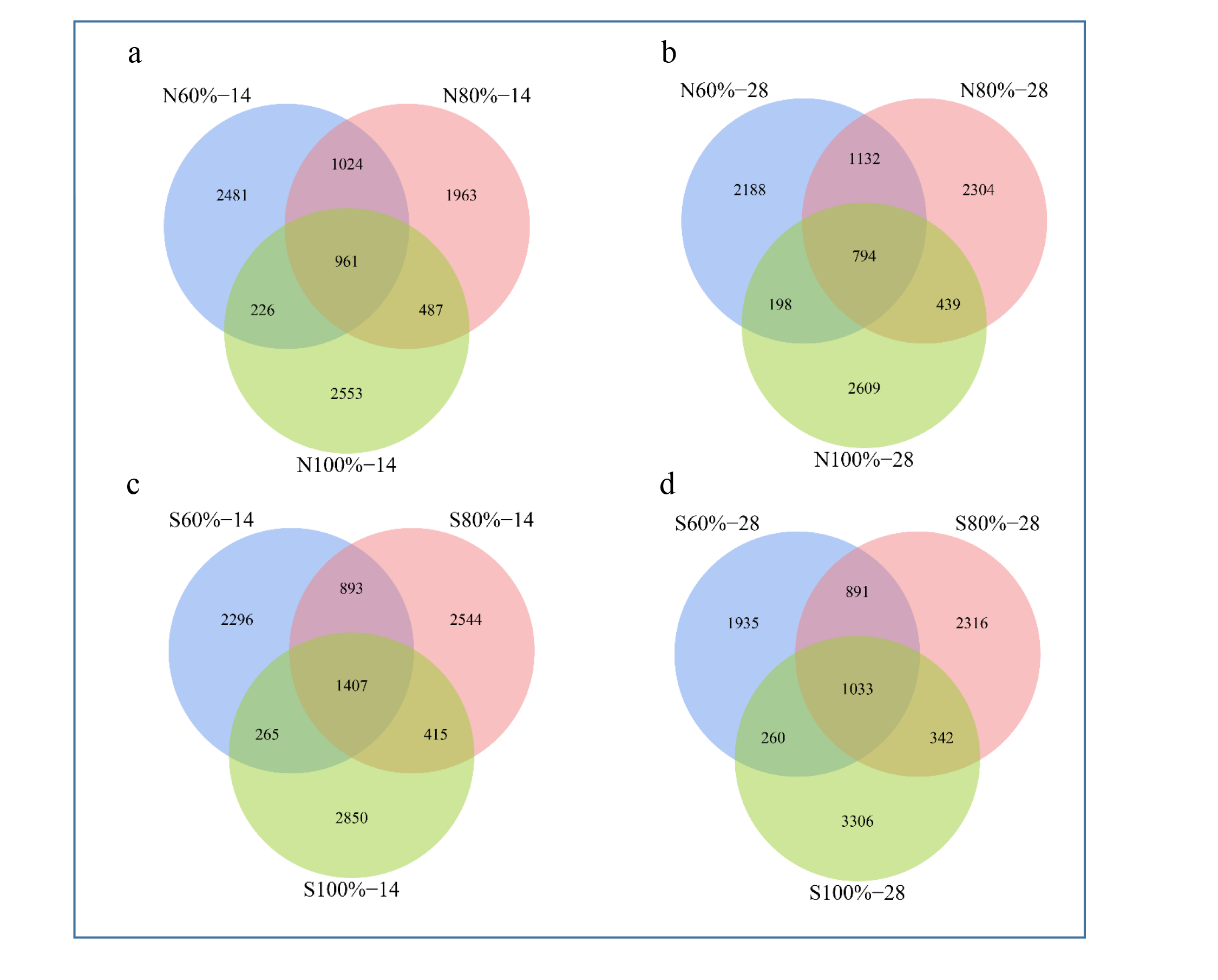


Figure S2 The venn diagram of the ASVs on days 14 and 28 in the clay and sandy soil. (a) Venn diagram of ASVs on days 14 in the clay soil. (b) Venn diagram of ASVs on days 28 in the clay soil. (c) Venn diagram of ASVs on days 14 in the sandy soil. (d) Venn diagram of ASVs on days 28 in the sandy soil.


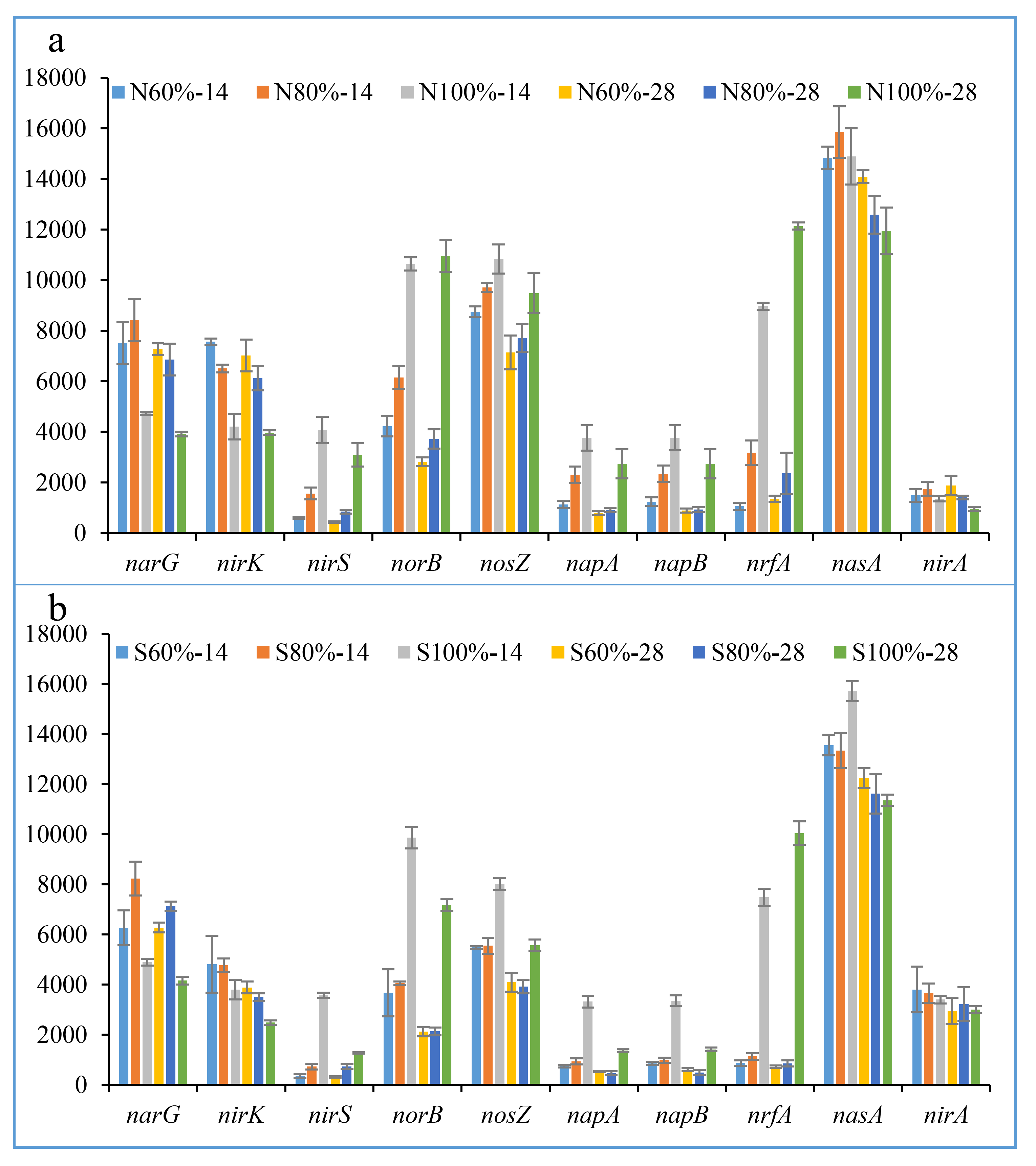


Figure S3 Enriched KEGG genes according to the PICRUSt2 analysis. The numerical value shows PICRUSt2 gene counts. The N60%, N80%, and N100% refer to the clay soil with 60%, 80%, and 100% filed water capacities, respectively. The S60%, S80%, and S100% refer to the sandy soil with 60%, 80%, and 100% filed water capacities, respectively. The number following the percentage indicates the number of days.


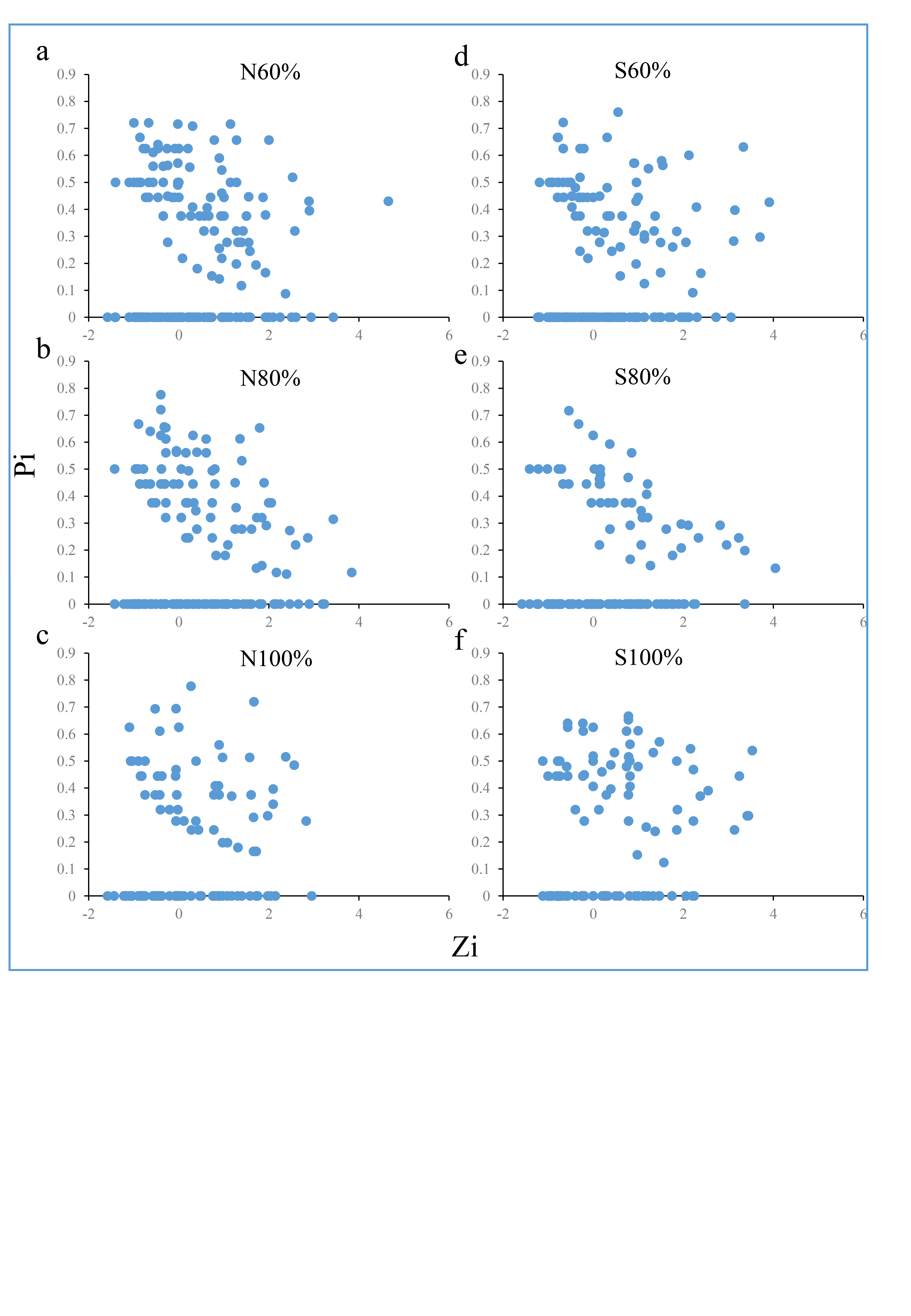


Figure S4 The within-module connectivity (Zi) and among-module connectivity (Pi) values of ASVs. The N60%, N80%, and N100% refer to the clay soil with 60%, 80%, and 100% filed water capacities, respectively. The S60%, S80%, and S100% refer to the sandy soil with 60%, 80%, and 100% filed water capacities, respectively.

Table S1 The original physical and chemical properties of the clay and sandy soils

|  | pH | EC | organic matter | available phosphorus | available potassium | total nitrogen |
| --- | --- | --- | --- | --- | --- | --- |
| clay soil | 6.84±0.05 | 246±7 | 10.49±0.40 | 30.65±0.56 | 428±12.22 | 0.76±0.05 |
| sandy soil | 6.45±0.07 | 84±7.09 | 20.93±0.94 | 61.22±1.02 | 122±8.08 | 1.17±0.06 |

|  | Observed ASV | Chao1 | ACE | Shannon |
| --- | --- | --- | --- | --- |
| N-0 | 2640.33 | 2672.61 | 2665.67 | 7.20 |
| S-0 | 2635.67 | 2665.22 | 2664.83 | 7.19 |

Table S2 The alpha index of N-0 and S-0
